# The genome of the protozoan parasite *Cystoisospora suis* and a reverse vaccinology approach to identify vaccine candidates^☆^

**DOI:** 10.1016/j.ijpara.2016.11.007

**Published:** 2017-03

**Authors:** Nicola Palmieri, Aruna Shrestha, Bärbel Ruttkowski, Tomas Beck, Claus Vogl, Fiona Tomley, Damer P. Blake, Anja Joachim

**Affiliations:** a Institute of Parasitology, Department of Pathobiology, University of Veterinary Medicine, Veterinärplatz 1, A-1210 Vienna, Austria; b Institute of Animal Breeding and Genetics, Department of Biomedical Sciences, University of Veterinary Medicine, Veterinärplatz 1, A-1210 Vienna, Austria; c Department of Pathology and Pathogen Biology, Royal Veterinary College, Hatfield, Hawkshead Lane, North Mymms AL9 7TA, UK

**Keywords:** Apicomplexa, Coccidia, Vacceed, Transcriptome, Illumina sequencing, Antigens

## Abstract

Vaccine development targeting protozoan parasites remains challenging, partly due to the complex interactions between these eukaryotes and the host immune system. Reverse vaccinology is a promising approach for direct screening of genome sequence assemblies for new vaccine candidate proteins. Here, we applied this paradigm to *Cystoisospora suis*, an apicomplexan parasite that causes enteritis and diarrhea in suckling piglets and economic losses in pig production worldwide. Using Next Generation Sequencing we produced an ∼84 Mb sequence assembly for the *C. suis* genome, making it the first available reference for the genus *Cystoisospora*. Then, we derived a manually curated annotation of more than 11,000 protein-coding genes and applied the tool Vacceed to identify 1,168 vaccine candidates by screening the predicted *C. suis* proteome. To refine the set of candidates, we looked at proteins that are highly expressed in merozoites and specific to apicomplexans. The stringent set of candidates included 220 proteins, among which were 152 proteins with unknown function, 17 surface antigens of the SAG and SRS gene families, 12 proteins of the apicomplexan-specific secretory organelles including AMA1, MIC6, MIC13, ROP6, ROP12, ROP27, ROP32 and three proteins related to cell adhesion. Finally, we demonstrated in vitro the immunogenic potential of a *C. suis*-specific 42 kDa transmembrane protein, which might constitute an attractive candidate for further testing.

## Highlights

• The draft genome of *Cystoisospora suis* is ∼84 Mb with > 11,000 protein-coding genes.
• Vacceed identified 1,168 candidates including apical complex proteins and surface antigens.
• 220 of these candidates are specific to apicomplexans and highly expressed in merozoites.
• Analysis of a selected *C. suis*-specific transmembrane protein confirmed the usefulness of reverse vaccinology.

## Graphical abstract

**Figure:**
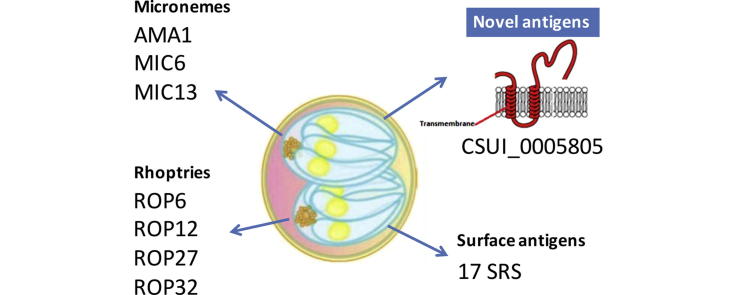

## 1 Introduction

*Cystoisospora suis* (syn. *Isospora suis*) is a protozoan parasite of the phylum Apicomplexa (subclass Coccidia, order Eucoccidiorida, family Sarcocystidae). This phylum contains almost exclusively obligate endoparasites of animals, including species of great medical and veterinary relevance such as *Plasmodium falciparum* and *Toxoplasma gondii*. According to recent reevaluations of the coccidian phylogeny (Ogedengbe et al., 2015, Samarasinghe et al., 2008), the position of *C. suis* in the family Sarcocystidae constitutes an outgroup of the cluster containing the genera *Neospora*, *Hammondia* and *Toxoplasma*. The closest outgroup genus of *C. suis* in the family Sarcocystidae is *Sarcocystis*, while the closest outgroup family of Sarcocystidae is Eimeridae, which contains the genus *Eimeria*.

*Cystoisospora suis* is responsible for neonatal porcine coccidiosis, a diarrheal disease of suckling piglets that causes significant economic losses in swine production worldwide (Lindsay et al., 1992, Meyer et al., 1999, Mundt et al., 2005, Aliaga-Leyton et al., 2011, Shrestha et al., 2015) (Torres, A., 2004. Prevalence survey of *Isospora suis* in twelve European countries. Proceedings of the 18th IPVS Congress, Hamburg, Germany, p. 243). The disease is commonly controlled with the triazinone toltrazuril, but drug costs and pressure to reduce the use of drugs in livestock production are increasing. Furthermore, resistance to toltrazuril has been described among parasites of the genus *Eimeria* (Stephen et al., 1997) and must be considered likely for *C. suis*. To date, no other drugs have been shown to be effective if administered in a way that is compatible with field use (Joachim and Mundt, 2011).

Alternative options for the control of this pathogen include vaccination. Immunological control measures (active or passive vaccination) commonly provide longer lasting protection than chemotherapeutic interventions and do not leave chemical residues in the host or the environment. Vaccine development for apicomplexan parasites has been hindered in part by their relatively complex life cycles and lack of in vitro and in vivo models for screening. Moreover, many apicomplexans have proved capable of evading immune killing by targeting immunoprivileged sites or through extensive antigenic diversity (Stanisic et al., 2013). Vaccination against some apicomplexan parasites such as the *Eimeria* spp. that infect chickens has been possible using formulations of live unmodified or attenuated parasites, but vaccine production requires passage and amplification in live animals with implications for cost, biosafety and animal welfare (Blake et al., 2015). For other protozoan parasites, success has been harder to achieve. However, progress towards antigen identification that could lead to development of recombinant or vectored vaccines has improved for several coccidian parasites in recent years (McAllister, 2014).

To date, no vaccine has been developed against *C. suis* although previous studies have shown that cellular and humoral immune responses are induced upon infection (Taylor, J., 1984. Immune Response of Pigs to *Isospora suis* (Apicomplexa, Eimeriidae), PhD Thesis, Auburn University, USA; Worliczek et al., 2010a, Schwarz et al., 2013, Gabner et al., 2014) and that superinfection of sows ante partum with high doses of oocysts can confer partial maternal protection (Schwarz et al., 2014). Since the use of live, virulent vaccines in large amounts is not practical and attenuated lines are not currently available for *C. suis*, a systematic search for proteins with antigenic properties is required to find appropriate vaccine candidates for testing and antigenic characterisation. A key step towards the identification of appropriate antigens for many apicomplexans has been the availability of genomic data, urging the development of a *C. suis* genome sequence assembly.

The approach of finding vaccine candidates using a genome sequence has been termed “reverse vaccinology”. This strategy has become a powerful way to identify proteins that can elicit an antigenic response with relevance to host/pathogen interaction. Reverse vaccinology is based on in silico screening of protein sequences to search for motifs and structural features responsible for inducing an immune response. Examples include transmembrane domains, signal peptides for excretion or surface membrane targeting and binding sites for Major Histocompatibility Complex (MHC) proteins. While this method has been successfully applied in bacterial pathogens (i.e. Pizza et al., 2000), only a few studies have been performed on eukaryotic pathogens. Examples include the apicomplexan species *T. gondii* (Goodswen et al., 2013, Goodswen et al., 2014b) and *Neospora caninum* (Goodswen et al., 2014a), as well as helminths such as *Schistosoma* (de Oliveira Lopes et al., 2013). Recently, the program Vacceed (Goodswen et al., 2014c) was developed, providing a high-throughput pipeline specifically designed to identify vaccine candidates in eukaryotic pathogens. This program was tested on the coccidian *T. gondii*, where it showed an accuracy of 97% in identifying proteins that corresponded to previously validated vaccine candidates (Goodswen et al., 2013).

In this work, we applied the reverse vaccinology paradigm to identify potential vaccine candidates in *C. suis*. To accomplish this, we used Illumina Next Generation Sequencing technology to sequence the *C. suis* genome and annotate protein-coding genes by combining ab initio and orthology predictions with gene models derived from a *C. suis* merozoite RNA-Seq library. Additionally, the annotation was manually curated at single gene resolution, greatly enhancing the quality of the gene models. Vacceed was then applied to perform a genome-wide screen for potential immunogenic proteins and identified 1,168 proteins with a high immunogenicity score. Finally, we validated the immunogenic potential of a *C. suis*-specific 42 kDa transmembrane protein (CSUI_005805) by performing an independent immunoblot analysis using positive polyclonal sera from infected piglets. These results show how reverse vaccinology, combined with comparative genomics and transcriptomics, can be applied to a eukaryotic pathogen to guide the identification of novel vaccine candidates as a starting point to develop a vaccine against *C. suis*. Moreover, the *C. suis* genome represents the first genomic sequence available for a member of the *Cystoisospora* group and it might serve as a reference for future studies involving *Cystoisospora* spp.

## 2 Materials and methods

### 2.1 Parasite material

For preparation of genomic DNA for sequencing, *C. suis* oocysts (strain Wien I) were isolated from experimentally infected piglets, left to sporulate in potassium bichromate and purified using a caesium chloride gradient (Worliczek et al., 2010b). DNA was extracted from 2.5 × 10^6^ washed and pelleted sporulated oocysts using a Peqlab Microspin tissue DNA kit (Peqlab, Erlangen, Germany) following the manufacturer’s instructions. RNAse A digestion was performed on the DNA before final purification. *Cystoisospora suis* merozoites were maintained in IPEC-J2 cells as described earlier (Worliczek et al., 2013). Free merozoites (8 × 10^6^) were harvested by collecting supernatant 6 days p.i. and purified on a Percoll® density gradient. Purified merozoites were then filtered through Partec CellTrics® disposable filters (50 μm), washed twice with PBS and pelleted by centrifugation at 1000*g* for 10 min. Total RNA was extracted from purified pelleted merozoites using a QIAamp RNA blood mini kit (Qiagen, Germany) according to the manufacturer’s instructions and was quantified using a spectrophotometer (NanoDrop 2000, Thermo Fisher Scientific, USA).

### 2.2 Genome assembly

A total of 80 million paired-end 100 bp reads were generated from an Illumina HiSeq 2000 platform (Source BioScience, UK) (reads were deposited at the Short Reads Archive – accession number **SRS1672208**). A genome sequence was assembled into contigs with CLC Genomics Workbench version 7.5 (CLC Bio-Qiagen, Aarhus, Denmark) using default parameters. In this study, we did not attempt to assemble chromosomes, as the main interest was in identifying proteins to screen for immunogenic features. To remove possible contaminants, we aligned the contigs against the non-redundant (nr) RefSeq protein database using BLASTN (E < 10^−5^) with default parameters and removed all contigs with a hit to non-apicomplexan organisms. Alignment of *C. suis* contigs to *N. caninum* and *T. gondii* genomes was performed with PROmer version 3.0.7 (Kurtz et al., 2004) (default parameters). Repetitive content was computed using RepeatMasker version 4.0.5 (parameters –species “toxoplasma apicomplexa”).

### 2.3 Genome annotation

Genes were annotated using Maker version 2.31.8 (Cantarel et al., 2008), which is a pipeline that combines different annotation tracks into a final set of gene models. Each annotation track was produced using the following programs:

#### 2.3.1 Augustus

Augustus is an ab initio gene predictor that can be trained with an accurate set of gene models, if available. To construct the training set we started from the Cufflinks gene track (described in more detail in Section 2.3.4), as it was based on the most accurate evidence for transcription, namely RNA-Seq data. Transcript sequences from the Cufflinks track were given to ORFPredictor version 2.3 (Min et al., 2005) (parameters strand ‘+’) to predict the location of each Coding Sequence (CDS). Transcripts were then filtered according to the following criteria: (i) transcripts with incomplete CDS were removed; (ii) in the case of multiple isoforms, the isoform with the longest CDS was retained; (iii) genes without introns were removed; (iv) genes with at least one exon made entirely of untranslated regions (UTRs) were removed; (v) genes separated by at least 500 bp from the previous or next gene were retained; (vi) genes with containing ambiguous nucleotides (Ns) in the upstream or downstream 500 bp flanking regions were removed. The resulting gene set was used to train Augustus version 3.01 (Stanke et al., 2006) to generate a new species model that was provided as a species parameter for Augustus (default parameters) to predict gene locations on the contigs and generate the final track.

#### 2.3.2 Snap

Snap is an ab initio gene predictor. We used the same training set constructed for Augustus to train Snap version 2006-07-28 (Korf, 2004) and generate the parameters file that was provided to Snap (default parameters) together with the contigs to produce the gene track.

#### 2.3.3 Exonerate

Exonerate is a homology based gene predictor which generates gene models based on the assumption that gene sequence and structure is conserved among closely related species. To create this gene track, protein sequences from *Eimeria tenella*, *N. caninum*, *Hammondia hammondi* and *T. gondii* were downloaded from ToxoDB (Gajria et al., 2008) release 24 and aligned to the contigs with Exonerate version 2.2.0 (parameters -m protein2genome –softmasktarget –percent 20 –showtargetgff –bestn 1).

#### 2.3.4 Cufflinks

Cufflinks is an evidence-based gene predictor which constructs gene models from RNA-Seq data. Total RNA was extracted from merozoites harvested from an in vitro culture (Worliczek et al., 2013) 6 days p.i. and sent for sequencing to GATC Biotech AG (Constance, Germany) using an Illumina sequencer HiSeq 2500 to generate paired-end reads of 100 bp (reads were deposited at the Short Reads Archive – accession number **SRS1683974**). A combined reference including the pig and *C. suis* genome was created, as the raw reads were likely to contain residual pig RNAs from the cell culture from which the merozoites were extracted. The *Sus scrofa* genome version 10.2 was downloaded from Ensembl (www.ensembl.org) and concatenated to the *C. suis* contigs. Reads were mapped to this combined reference with TopHat version 2.1.0 (Kim et al., 2013) (parameters –library-type fr-firststrand) (see Supplementary Table S1 for RNA-Seq mapping statistics). Reads mapped to the pig genome were filtered out and the resulting BAM file was provided to Cufflinks version 2.2.1 (Trapnell et al., 2010) (parameters –library-type fr-firststrand) to reconstruct gene models. Cufflinks additionally computes the expression level of each gene using the standard FPKM measure (Fragments per Kilobase of Exon per Million reads mapped), which normalises the number of reads mapped to a gene by gene length and the total number of reads in the dataset (for more details see Mortazavi et al. (2008)). Transcripts with low expression (FPKM < 1) were removed and the output was converted to GFF3 format using the cufflinks2gff3 script from the Maker pipeline. Additionally, junctions derived from TopHat were converted to GFF3 with the tophat2gff3 script from Maker and added to the Cufflinks GFF3 to get the final track.

After generating the four gene tracks, Maker was run to generate the uncurated Maker track with the following parameters that were provided in the configuration file:

genome = FASTA with contigs
augustus_species = species name (*cystoisospora*) from the Augustus training
snaphmm = HMM parameter file from the Snap training
protein = FASTA with proteins from *E. tenella*, *N. caninum*, *H. hammondi* and *T. gondii*
Furthermore, these additional parameters were specified: (est2genome = 1, protein2genome = 1, pred_flank = 500, alt_splice = 0, single_exon = 1, correct_est_fusion = 1).

### 2.4 Manual curation

The BAM file from TopHat, the four gene tracks (Augustus, Snap, Exonerate, Cufflinks) and the uncurated Maker gene track were loaded into the Integrative Genomics Viewer (IGV) (Thorvaldsdottir et al., 2013). Each gene was independently curated in the following way: in the case of incongruences among tracks, priority was given to the Cufflinks evidence, followed by Exonerate, Augustus and Snap. We decided to prioritise Augustus over Snap, as Snap produced a high number of very short terminal exons, which we cautiously regarded as unreliable. Gene models corresponding to lowly expressed genes were resolved only in some cases, as their exon–intron structure was often very fragmented. The genome and annotation of *C. suis* are available in the National Center for Biotechnology Information, (NCBI, USA) database under the accession number **PRJNA341953**.

### 2.5 Evaluation of annotation quality

To assess the quality of the annotation, cuffcompare (Trapnell et al., 2010) was run to compute the fraction of final gene models confirmed by each type of evidence (Cufflinks, Exonerate, Augustus, Snap). To evaluate the completeness of the annotation, the core eukaryotic proteins from Parra et al. (2007) were downloaded from http://korflab.ucdavis.edu/datasets/cegma/core/core.fa and aligned to the contigs with BLASTP (E < 10^−5^, query coverage ⩾ 30%), retaining only the best hit. Eukaryotic core proteins that did not align to contigs (unaligned proteins) were further analysed to check for their presence in the RNA-Seq dataset using the following steps: (i) RNA-Seq reads from merozoites were de novo assembled into transcripts using Oases (Schulz et al., 2012) with default parameters; (ii) unaligned proteins were aligned to the assembled transcripts with TBLASTN (E < 10^−5^).

### 2.6 Functional annotation

Functional annotation was performed with Blast2GO (Conesa et al., 2005) version 3.1.3 on the protein sequences using the following steps: (i) blastp-fast (E < 10^−5^) was run against the local version of the nr database downloaded on 01 December, 2015 and used to generate a first version of the Gene Ontology (GO) functional annotation; (ii) InterProScan (Jones et al., 2014) was run and results were merged to the initial GO annotation in order to extend it, (iii) ANNEX (Myhre et al., 2006) was run to further augment the GO annotation. The final functional annotation also allowed the identification of additional transposable elements that were not found by RepeatMasker.

### 2.7 Detection of vaccine candidates

Initially, 403 protein sequences containing unknown amino acids (‘X’) were filtered out. The software Vacceed (Goodswen et al., 2014c) was used to identify potential immunogenic proteins. Vacceed implements a machine learning approach that combines independent sources of evidence for immunogenic features computed by different tools: WoLf PSORT (Horton et al., 2007), SignalP (Petersen et al., 2011), TargetP (Emanuelsson et al., 2000), Phobius (Kall et al., 2007), TMHMM (Sonnhammer et al., 1998), MHC-I Binding and MHC-II Binding. The program finally assigns a score between 0 and 1 to each protein to rank the protein from low immunogenicity (score = 0) to high (score = 1). We excluded MHC-II Binding in our analyses as data about MHC-II allele binding affinity is unavailable for the pig. Moreover, prior to running Vacceed the tool MHC-I Binding was trained using known immunogenic proteins as input. Since no known immunogenic proteins were available for *C. suis*, we used *T. gondii* proteins from Goodswen et al. (2014c) to train MHC-I Binding for affinity against Swine Leukocyte Antigens (SLA) alleles, as *T. gondii* was the closest relative for which a dataset of immunogenic and non-immunogenic proteins was available. Moreover, as the computational burden of the MHC-I Binding predictor was very high, we divided the Vacceed analysis into two steps: (i) we ran Vacceed on all the protein sequences using each tool except MHC-I Binding and ranked the proteins according to score; (ii) we selected all proteins with a score > 0.75 and reran Vacceed on this subset using all the tools, including MHC-I Binding. Finally, from the score distribution obtained after step ii, we selected as candidates only proteins with a score ⩾ 0.998, corresponding to the top 25% of the score distribution.

### 2.8 Ortholog assignment for vaccine candidate genes

To assign orthologs of *C. suis* genes in the other coccidian species, protein sequences from *E. tenella*, *Sarcocystis neurona*, *N. caninum*, *H. hammondi* and *T. gondii* were downloaded from ToxoDB (Gajria et al., 2008) release 24 and clustered with the *C. suis* proteins using the Orthology MAtrix (OMA) software (Altenhoff et al., 2013) with parameters LengthTol = 0.30. For genes for which OMA did not detect an ortholog in any species, we manually screened for its presence in the ToxoDB database by looking at the section “Orthologs and Paralogs within ToxoDB” within the gene entry.

### 2.9 Two-dimensional (2D) gel electrophoresis

For 2D gel electrophoresis, 6 × 10^6^ merozoites harvested from cell culture were purified and concentrated as described above and directly dissolved in DIGE buffer (7 M urea, 2 M thiuourea, 4% CHAPS, 1% (w/v) DTT, 20 mM Tris) and centrifuged again at 20,000*g* for 10 min at 4 °C. The protein concentration of each lysate was determined by Bradford assay (Bradford, 1976). For separation in the first dimension, an aliquot of 40 μg of protein was diluted in 300 μl of rehydration solution (8 M urea, 2% (w/v) CHAPS, 12.7 mM DTT, 2% immobilised pH gradient (IPG) buffer, 0.002% (w/v) bromophenol blue) and used to rehydrate 13 cm IPG strips with a non-linear gradient pH 3–10 (Immobiline, GE Healthcare Life Sciences, Uppsala, Sweden) for 18 h at room temperature. Isoelectric focusing (IEF) was carried out (300 V ascending to 3,500 V for 90 min, followed by 3,500 V for 18 h) using a Multiphor II electrophoresis chamber (GE Healthcare Life Sciences). After IEF, the IPG strips were equilibrated with 10 mg/ml of DTT in equilibration buffer (6 M urea, 30% glycerol, 2% (w/v) SDS, 0.002% (w/v) bromophenol blue, 1.5 M Tris–HCl) for 20 min and further incubated in the same buffer for another 20 min, replacing DTT with 25 mg/ml of iodoacetamide. The IPG strips were then washed with deionised water. In the second dimension, SDS–PAGE was performed using vertical slab gels (1.5 mm; T = 12%, C = 2.6%, 1.5 M Tris–HCl, 10% SDS) under reducing conditions at 15 mA for 15 min, followed by 25 mA in a Protean II electrophoresis chamber (Bio-Rad Laboratories, Hercules, USA). Each gel was stained with silver (Blum and Gross, 1987) and scanned using the program ImageMaster™ 2D platinum v.7.0.

### 2.10 Immunoblot analysis

Proteins separated by 2D gel electrophoresis were transferred onto a Trans-Blot® (Bio-Rad) nitrocellulose (NC) membrane (0.45 μm) for 150 min at 35 V, 150 mA and 6 W on a Nova Blot semi-dry transfer system (Pharmacia Biotech, USA). The membranes were dried overnight in the dark at room temperature. The next day, blots were blocked for 1 h using 2% (w/v) BSA. After three washes with TTBS buffer (100 mM Tris, 0.9% NaCl, 0.1% Tween 20), the blots were incubated with porcine anti-*C. suis* serum (Schwarz et al., 2013) (having an IFAT antibody titer ⩾ 10,000) diluted in TTBS buffer (1:300) under gentle agitation at room temperature for 30 min. After rinsing in TTBS for 15 min, blots were exposed to biotinylated goat anti-pig IgG (Vector laboratories, USA; 1:500 dilution) as secondary antibody for 30 min at room temperature, incubated with ABC solution (Vector Laboratories) and finally detected by 3,3′-5,5′-tetramethylbenzidine (TMB). Pre-colostral sera from non-infected piglets served as negative controls (Schwarz et al., 2013).

### 2.11 Mass spectrometry

The spot of interest from 2D gel was excised, washed, destained (Gharahdaghi et al., 1999), reduced with DTT and alkylated with iodoacetamide (Jimenez et al., 2001). In-gel digestion was performed with trypsin (Trypsin Gold, Mass Spectrometry Grade, Promega, Fitchburg, USA) according to Shevchenko et al. (1996) with a final trypsin concentration of 20 ng/μl in 50 mM aqueous ammonium bicarbonate and 5 mM calcium chloride. Dried peptides were reconstituted in 10 μl of 0.1% (v/v) trifluoroacetic acid (TFA). Nano-HPLC separation was performed on an Ultimate 3000 RSLC system (Dionex, Amsterdam, Netherlands). Sample pre-concentration and desalting were accomplished with a 5 mm Acclaim PepMap μ-precolumn (300 μm inner diameter, 5 μm particle size, and 100 Å pore size) (Dionex) with a flow rate of 5 μl/min using a loading solution (2% Acetonitrile (ACN) in 0.05% aqueous TFA). The separation was performed on a 25 cm Acclaim PepMap C18 column (75 μm inner diameter, 3 μm particle size, and 100 Å pore size) with a flow rate of 300 nl/min. The gradient started with 4% B (80% ACN with 0.1% formic acid) and increased to 35% B over 120 min. It was followed by a washing step with 90% B for 5 min. Mobile Phase A consisted of mQH2O with 0.1% formic acid. The injection volume was 1 μl partial loop injection mode. For mass spectrometric analysis, the LC was directly coupled to a high-resolution quadrupole time of flight mass spectrometer (Triple TOF 5600+, Sciex, USA) was used. For information-dependent data acquisition (IDA runs), MS1 spectra were collected in the range 400–1500 *m*/*z*. The 25 most intense precursors with charge state 2–4 which exceeded 100 counts per second were selected for fragmentation, and MS2 spectra were collected in the range 100–1800 *m*/*z* for 110 ms. The precursor ions were dynamically excluded from reselection for 12 s. The nano-HPLC system was regulated by Chromeleon 8.8 (Dionex) and the MS by Analyst Software 1.7 (Sciex). Processed spectra were searched via the software Protein Pilot (Sciex) against *T. gondii* extracted from the UniProt_TREMBL database as well as against our *C. suis* protein database using the following search parameters: Global modification: Cysteine Alkylation with Iodacetamide, Search effort: rapid, FDR analysis: Yes. Proteins with more than two matching peptides at > 95% confidence were selected.

## 3 Results

### 3.1 Genome assembly

**Fig. 1:**
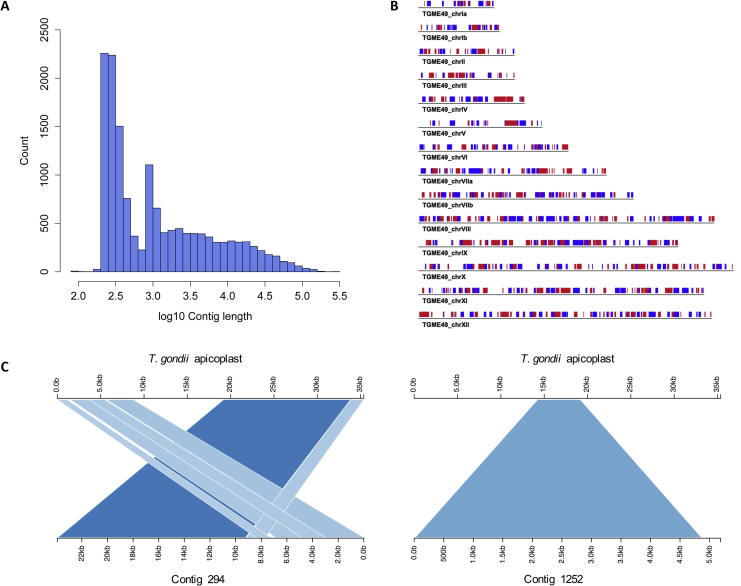
General contig-based statistics for the *Cystoisospora suis* genome. (A) Distribution of *C. suis* contig lengths. (B) Alignment of *C. suis* contigs to the chromosomes (chr) of *Toxoplasma gondii*; contigs are colored according to orientation (blue = plus, red = minus). Only 27.8% of the contigs base pairs align to *T. gondii*. (C) BLAST alignment of the *T. gondii* apicoplast to the two best matching *C. suis* contigs (different colors are used for the bands for graphical clarity). (For interpretation of the references to colour in this figure legend, the reader is referred to the web version of this article.)

We generated a total of 14,776 contigs from 80 M paired-end Illumina reads (see Supplementary Table S2 for mapping statistics). The assembly had an N50 of 29,979 bp, with minimum and maximum contig lengths of 89 bp and 285,055 bp. (N50 is the largest length L such that 50% of all nucleotides are contained in contigs of a size that is at least L.) A graphical distribution of contig lengths is shown in Fig.1A. To exclude bacterial and other contaminations, we aligned the contigs to the nr database and retained 14,630 contigs without matches to other organisms outside the Apicomplexa and covering a length of 83.6 Mb. We used this set of contigs for the remainder of the analyses. In Table 1 we report a comparison about genome size and GC content between *C. suis* and other coccidians.

**Fig. 2:**
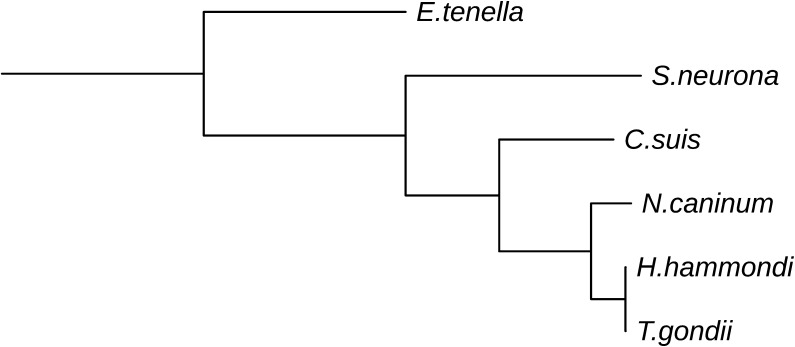
Phylogenetic tree showing the relationships among the coccidian species analysed in this study (*Toxoplasma gondii, Hammondia hammondi, Neospora caninum, Cystoisospora suis, Sarcocystis neurona, Eimeria tenella*) reconstructed using the glyceraldehyde 3 phosphate dehydrogenase 1 (GAPDH1) gene. The *T. gondii* protein TGME49_269190 was aligned with BLASTP (default parameters) to the proteomes of the other species and the best hit was retained. Protein sequences were aligned with PRANK (Loytynoja and Goldman, 2005) (parameters –f = phylipi –protein) and a maximum likelihood tree was constructed using proml from the PHYLIP package (Felsenstein, J. 2005. PHYLIP (Phylogeny Inference Package) version 3.6. Distributed by the author. Department of Genome Sciences, University of Washington, Seattle, USA) with parameters “Speedier but rougher analysis? No, not rough”.

To investigate evolutionary divergence from other coccidians we aligned the *C. suis* contigs to the genomes of the closest relatives, *T. gondii* and *N. caninum,* in the coccidian phylogeny (Fig. 2). Only 27.8% and 28.1% of the *C. suis* bases aligned, respectively, to *T. gondii* and *N. caninum* (Fig.1B). This translated into 722 *C. suis* contigs successfully aligned to *T. gondii*, covering a total of 3,143 *C. suis* genes, and 733 contigs aligned to *N. caninum*, including 3,195 *C. suis* genes.

The GC content of the *C. suis* genome was 50.0%, similar to that of other coccidian species (Table 1). Among repetitive sequences 5.07% were simple repeats, 1.84% low complexity regions and 0.02% small RNAs. We identified 93 genes associated with transposons distributed as follows: 58 genes from the ty3-gypsy subclass, six tf2 retrotransposons, one FOG transposon and 28 genes encoding for retrotransposon accessory proteins, such as gag/pol.

Most apicomplexan parasites possess a special organelle called the apicoplast (Sato, 2011), which is a plastid acquired through secondary horizontal transfer from an algal ancestor and has functions related to secondary metabolism. We attempted to identify contigs corresponding to the apicoplast genome. By aligning the *T. gondii* apicoplast sequence to the *C. suis* contigs using BLASTN (default parameters), we found that 89.4% of the *T. gondii* sequence mapped to three *C. suis* contigs. Most of the apicoplast sequence was covered by contig 294 (∼24 kb) (Fig.1C), with additional short fragments located on contigs 1252 (Fig.1C) and 6453 (not shown as it contained redundant sequence overlapping with contig 1252). Gene annotation confirmed the apicoplast origin of these contigs, with a total of 25 protein-coding genes all well conserved with *T. gondii*. The genes encode for 14 ribosomal proteins, the elongation factor *tu*, orf c, d, e, f, the caseinolytic protease C, four RNA polymerases and the cysteine desulfurase activator complex subunit. Four of the ribosomal proteins contain a premature stop codon, which might suggest that they are pseudogenes. Finally, five *T. gondii* genes did not have an ortholog in the *C. suis* apicoplast. To further characterise apicoplast genes we looked at RNA-Seq expression data from merozoites (Hehl et al., 2015), however we found none of the genes to be expressed in either *C. suis* or *T. gondii* at this stage. The complete list of apicoplast genes is available in Supplementary Table S3.

### 3.2 Genome annotation

To annotate protein-coding genes we applied the MAKER pipeline, as outlined in Section 2.3, followed by a manual curation of each individual gene. To quantify the effect of curation we computed the total number of genes, the percentage of exonic base pairs overlapping with exons generated from the RNA-Seq dataset (nucleotide level sensitivity – also described in Burset and Guigo (1996)) and the percentage of genes with UTRs before and after curation. The initial uncurated annotation contained 10,065 genes with a nucleotide level sensitivity of 68.8%, 5,553 genes with 5′ UTRs and 4,911 genes with 3′ UTRs. After curation, we obtained 11,572 genes, a nucleotide level sensitivity of 85.1%, 9,806 genes with 5′ UTRs and 8,485 genes with 3′ UTRs. These results showed that our curation greatly enhanced the quality of the annotation by increasing the concordance with transcriptional evidence and the number of genes with UTRs.

The total gene number appears to be higher in *C. suis* compared with other coccidians (Table 2), although lowly expressed genes might represent transcriptional noise and artificially increase the total gene count. To test this hypothesis, we removed genes with FPKM < 1, as this threshold is commonly used to distinguish transcriptional noise from real transcription. We detected 1,207 genes (10.4%) with FPKM < 1, implying that lowly expressed genes can only partially account for the higher number of genes in *C. suis* compared with other species.

To verify the completeness of our annotation, we checked for the presence of eukaryotic core genes that should be conserved in all eukaryotes (Parra et al., 2007). From 458 eukaryotic core genes, 396 (86%) were present in our gene catalogue. We looked for the presence of the missing genes in filtered contigs and in transcripts reconstructed de novo from the RNA-Seq dataset. Of the remaining 62 eukaryotic core genes, none were found in the filtered contigs and 16 were found in the de novo assembled transcripts. A total of 46 eukaryotic core genes was thus not found in our *C. suis* data. To shed light on whether these might constitute missing annotations or genuine losses, we looked for their presence in the proteomes of the coccidian species listed in Table 1. We found 29 genes present in at least one coccidian species. The remaining 17 genes were not found in any species, and thus were likely true gene losses in this clade (Supplementary Table S4). If this set of genes is excluded from the initial list of eukaryotic core genes a final completeness of (396 + 16)/(458 − 17) * 100 = 93% is obtained.

To further assess the quality of the annotation we assigned to each gene a binary vector summarizing the kind of evidence that was used to annotate it (transcriptional, orthology-based or ab initio). Generally, the most reliable source of annotation is transcription evidence, followed by orthology inference and ab initio predictions. Of a total of 11,572 genes, 10,452 (90.3%) were supported by transcription evidence (Cufflinks). Among the remaining 1,120 genes, 435 (3.8%) were supported by orthology evidence (Exonerate) and 338 (2.9%) only by ab initio predictors (either Augustus or Snap). Finally, 347 genes (3.0%) were only partially supported by any kind of evidence and would require further curation.

Next, we predicted CDS for 11,545 genes (99.8%) using ORFPredictor (Min et al., 2005). We classified genes according to the completeness of the CDS as follows: 7,801 genes (67.8%) had a complete CDS, i.e. with start and stop codon; 1,964 (17.0%) had only a start codon, 1,161 (10.0%) only a stop codon and 619 (5.4%) were CDS fragments, i.e. without start and stop codons. The complete annotation of *C. suis* including exons, introns, CDS and UTRs is available at the NCBI database under accession number **PRJNA341953**.

Finally, the phylogenetic distribution of *C. suis* genes was assessed using OrthoMCL and found that 6,387 (55.4%) of the protein coding genes were assigned to an ortholog group.

### 3.3 Detection of vaccine candidates

**Fig. 3:**
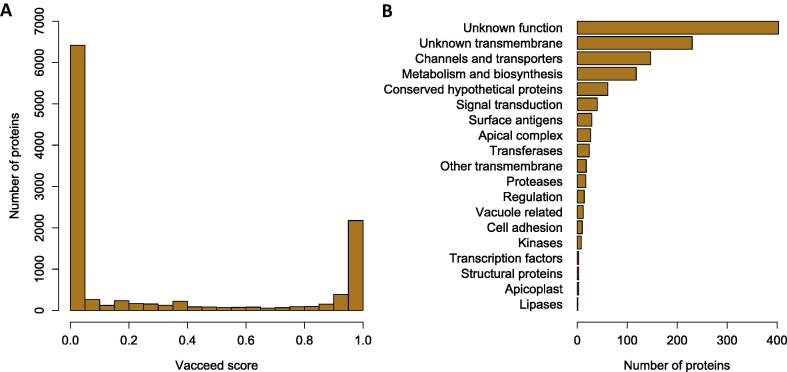
General statistics for the non-stringent set of vaccine candidates. (A) Distribution of Vacceed scores for *Cystoisospora suis* proteins using all the tools except Major Histocompatibility Complex (MH-I) binding (see Section 2.7). (B) Functional classification of vaccine candidates.

We applied the tool Vacceed to screen the *C. suis* proteome for vaccine candidates. Due to the high computation time of the MHC-I Binding tool, the Vacceed analysis was divided into two steps (see Section 2.7). Fig.3A shows the score distribution after the first step of Vacceed. This resulted in a clearly bimodal distribution from which 2,905 proteins with a score > 0.75 were selected. During the second step of Vacceed we refined this set including the MHC-I Binding tool and obtained 1,168 final candidate proteins (non-stringent set). By looking at the classification of candidates by functional class (Fig.3B), we observed that most of the proteins had no annotated function (403 proteins) or contained transmembrane domains but had unknown function (230 proteins). Among proteins with known function, the most abundant were channels and transporters (147 proteins), followed by proteins involved in metabolism and biosynthesis (118 proteins). Notable was the presence of apicomplexan-specific secretory organelles proteins (27 proteins) such as rhoptry kinases, microneme and dense granule proteins, which are involved in parasite motility, attachment, invasion and re-modelling of the intracellular parasite environment.

### 3.4 Contribution of immunogenic features

**Fig. 4:**
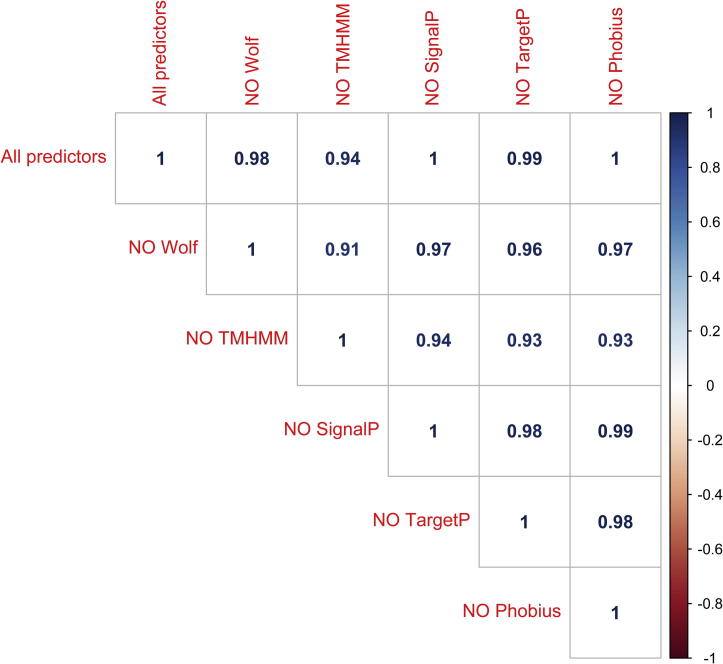
Correlation plot of Vacceed score distributions computed with different combinations of predictors. Each square contains the Pearson correlation coefficient between two Vacceed runs on all the *C. suis* proteins. (All predictors = Vacceed run using all predictors; NO WoLf = Vacceed run using all predictors except WoLf; other labels are similarly constructed).

Next, we wanted to establish the contribution of different immunogenic features in defining a protein as immunogenic, as Vacceed utilises different sources of evidence from various tools: WoLf PSORT to predict protein subcellular localisation; SignalP, TargetP, Phobius for detecting transmembrane domains; TMHMM and MHC-I Binding. For this analysis, we excluded MHC-I Binding due to its high computational burden. For each tool, Vacceed was run on the *C. suis* proteome using all other tools except for the tool in question (i.e. all tools except WoLf, then all tools except TMHMM and so on) and the score distributions were compared with the one computed using all tools (Fig. 4). All correlations were very high, showing that removing any one of the tools from Vacceed did not significantly affect the score distribution. However, it appeared that TMHMM was the program that contributed most to the immunogenic score since, when it was omitted, the correlation with the score computed with all tools was the lowest (*r* = 0.94).

### 3.5 Comparison with vaccine candidates in other coccidians

**Fig. 5:**
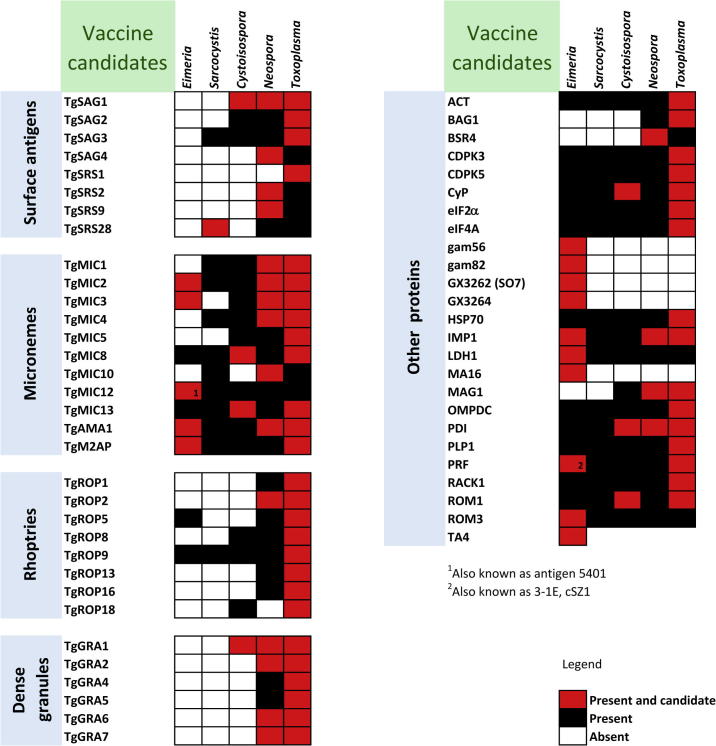
Orthology comparison of vaccine candidate proteins between *Cystoisospora suis* and other coccidian spp. *Hammondia hammondi* was excluded as no vaccination studies have been performed. Orthologs of *C. suis* genes were detected using the Orthology Matrix (Altenhoff et al., 2013) (see Section 2.8).

Vaccine candidates in coccidians can point to homologous proteins that may also induce immune protection against other coccidians. For this reason, we collected all tested vaccine candidates from an extensive screen of the published literature (Lillehoj and Lillehoj, 2000, Jenkins, 2001, Nishikawa et al., 2002, Bhopale, 2003, Elsheikha and Mansfield, 2004, Williams and Trees, 2006, Dlugonska, 2008, Jongert et al., 2009, Nam, 2009, Reichel and Ellis, 2009, Monney et al., 2011, Cui et al., 2012, Blake and Tomley, 2014, Hiszczynska-Sawicka et al., 2014, Lim and Othman, 2014, Monney and Hemphill, 2014) and from the VIOLIN database (Xiang et al., 2008) for *E. tenella*, *S. neurona*, *N. caninum* and *T. gondii*. No previously tested candidates were found for *H. hammondi*. We found 13 candidates for *E. tenella*, one for *S. neurona*, 19 for *N. caninum* and 43 for *T. gondii*, giving a union set of 58 total candidates (Fig. 5). These mostly included apicomplexan-specific secretory organelle proteins and surface antigens, which are known to be directly involved in the invasion process, but also a heterogeneous set of other proteins with no overrepresented function. Out of the 58 candidates, 34 were also present in the *C. suis* proteome and seven overlapped with our set of *C. suis* vaccine candidates, namely proteins orthologous to microneme proteins TgMIC8 and TgMIC13, the dense granule antigen TgGRA1, the surface antigen TgSAG1, the cyclophilin CyP, the immune mapped protein IMP1 and the protein disulphide isomerase PDI. This enrichment of previously described vaccine candidates in our Vacceed set was highly significant (one-tailed Fisher’s Exact Test – *P* = 2.2 × 10^−5^). Notably, most previously described rhoptry and dense granule candidate vaccine proteins were absent in *C. suis*, as they originated before the split of *T. gondii*, *H. hammondi* and *N. caninum*. On the other hand, many microneme proteins were present in *C. suis* but they were not classified as vaccine candidates in our study: three of those (orthologues of TgMIC2, TgMIC5, TgMIC12) had a Vacceed score that was only slightly lower than our threshold, while the remaining three (TgMIC1, TgMIC3, TgMIC4) had a very low score.

### 3.6 Evolutionary conservation of vaccine candidates

**Fig. 6:**
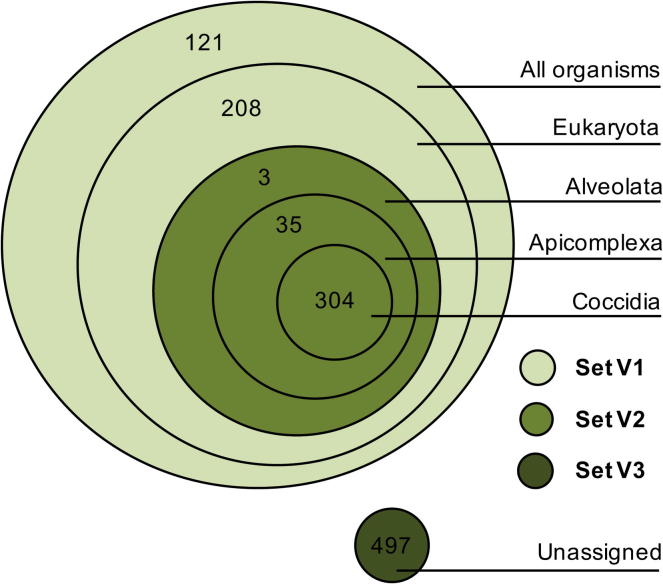
Phylogenetic distribution of the non-stringent set of vaccine candidates. Each circle contains the number of genes shared by that taxonomical group. See the Section 3.6 for the descriptions of the different sets (V1, V2 and V3).

Proteins that are phylogenetically restricted to *C. suis* or conserved only among closely related species might be more attractive candidates for experimental testing, since proteins with homologs and conserved epitopes in the host might induce an unwanted autoimmune response. To study the evolutionary conservation of vaccine candidates, we applied OrthoMCL and classified proteins according to taxonomic levels (Fig. 6). Approximately 28% of the proteins were very conserved in eukaryotes or shared by all organisms (set V1), another 29% were restricted to coccidians, apicomplexans or alveolates (set V2), while the largest fraction (43%) was not assigned to any ortholog group (set V3). If one would exclude the most conserved proteins (set V1) from in vitro testing, there is still a large set of candidates (839 proteins) to be investigated. By looking at GO functional enrichment of apicomplexan-specific and coccidian-specific proteins we observed a significant overrepresentation for “calcium ion binding” (*P* < 2.5 × 10^−7^) and “protein kinase activity” (*P* = 0.00067). Similarly, for coccidian-specific proteins the most prominent functional terms were “calcium ion binding” (*P* < 7.4 × 10^−6^) and “cyclic-nucleotide phosphodiesterase activity” (*P* = 0.00015).

### 3.7 Expression of vaccine candidates in merozoites

**Fig. 7:**
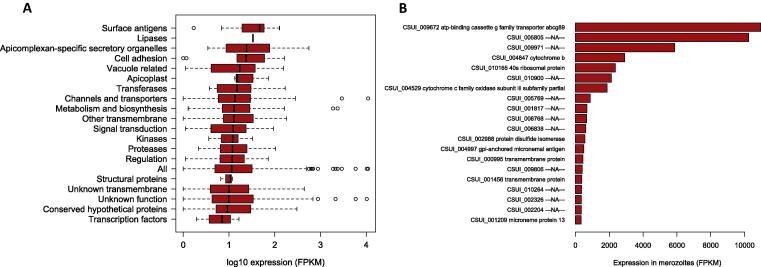
Gene expression statistics for the non-stringent set of vaccine candidates (A) Merozoite expression distribution of vaccine candidates classified by functional category. (B) Gene expression of the top 20 most expressed vaccine candidates. FPKM, Fragments per Kilobase of Exon per Million reads.

It is usually assumed that highly expressed genes are more likely to induce a sustained immunogenic response compared with lowly expressed ones (Flower et al., 2010, Goodswen et al., 2014a). To gain insight into the expression of genes encoding for immunogenic proteins we performed an RNA-Seq experiment using polyadenylated RNA purified from *C. suis* merozoites, as those constitute the primary intracellular reproductive stage of *C. suis* and interact directly with the host during invasion. Genes with functions related to invasion were the most highly expressed (Fig.7A) and included surface antigens, apicomplexan-specific secretory organelles, cell adhesion and motility, and parasitophorous vacuole-related genes. When looking at the total ranked set of candidates according to expression level (Fig.7B), we observed that more than 50% of the highly expressed candidates had unknown functions. Other proteins such as transporter abcg89, cytochrome b and c, were highly expressed and well characterised, but phylogenetically highly conserved and thus less suitable for further experimentation. Finally, 13 uncharacterised genes (unknown function or including generic transmembrane domains) with very high expression (FPKM > 30) and *C. suis* specificity might constitute attractive candidates for in vitro testing (Fig.7B – proteins annotated as “–NA–“ or “transmembrane protein”).

### 3.8 Stringent list of vaccine candidates

To produce a more stringent list of candidates, we selected from the 1,168 vaccine candidates only those proteins that were highly expressed in merozoites (FPKM > 30, which corresponds to the top 25% of the expression distribution of vaccine candidates, see Fig.7A – category “All”) and without orthologs outside the Apicomplexa. This resulted in a set of 220 proteins. These include 152 proteins with unknown function, of which 88 contain transmembrane domains. Additionally, there were 17 surface antigens related to the TgSAG/SRS gene families, 12 apicomplexan-specific secretory organelles proteins including orthologues of TgAMA1, TgMIC6, TgMIC13, TgROP6, TgROP12, TgROP27, TgROP32, nine proteins involved in metabolism and biosynthesis, seven channels and transporters proteins and three proteins related to cell adhesion. For the complete list of candidates see Supplementary Table S5.

### 3.9 Validation of vaccine candidates by immunoblot analysis

**Fig. 8:**
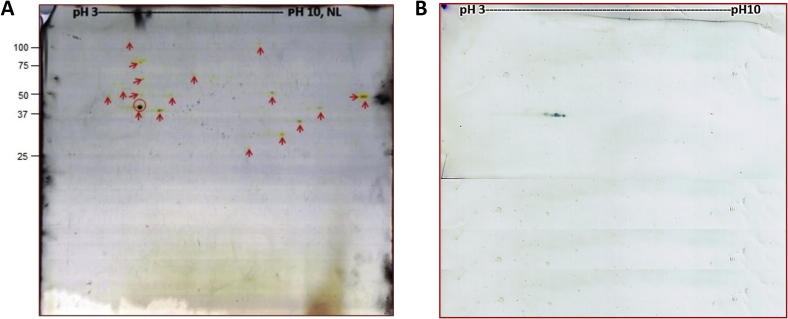
Protein blots of *Cystoisospora suis* merozoites lysates (A) Two-dimensional proteome map of *C. suis* merozoite lysates visualised using silver stain; arrows mark the detectable proteins; the circle marks the spots identified by western blot (B). (B) Two-dimensional immunoblot profile of *C. suis* merozoite lysates detected using positive sera from infected piglets. NL, non-linear.

**Fig. 9:**
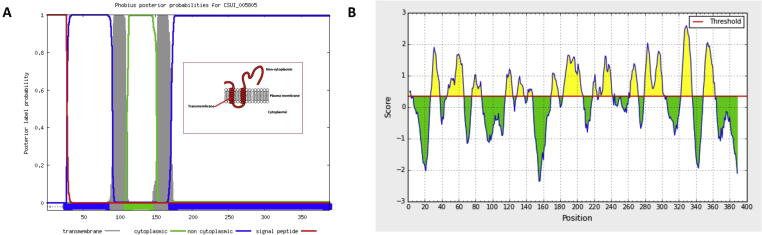
Structural information for the vaccine candidate protein CSUI_005805. (A) Domain prediction performed with the program Phobius on the protein CSUI_005805. Different colors indicate the regions of the protein in relationship to the cell membrane. The drawing in the red-bordered rectangle depicts a simplified representation of the protein structure. (B) B-cell epitope prediction performed with the Immune Epitope Database (IEDB) Analysis Resource tools on the protein CSUI_005805. The yellow peaks indicate the locations of the epitopes. For an explanation of the scoring system see Larsen et al. (2006). (For interpretation of the references to colour in this figure legend, the reader is referred to the web version of this article.)

To test whether vaccine candidate proteins interact with the host immune system and induce an immune response during *C. suis* infection, we performed a 2D immunoblot experiment using positive polyclonal sera from experimentally infected piglets. We resolved crude lysates from cultured *C. suis* merozoites using a broad range (pH 3–10) of 2D gels. These revealed 18 spots that were easily visualised by silver staining, likely corresponding to the most highly expressed proteins in the merozoite proteome (Fig.8A). To detect proteins that are recognised as antigenic by serum antibodies of infected hosts, we performed an immunoblot of the 2D gel probed with highly positive sera from experimentally infected piglets. This revealed one immuno-reactive spot (Fig.8B), whereas no reactive spots were detected in the immunoblot probed with precolostral sera (data not shown). Protein sequencing by mass spectrometry showed that the spot corresponded to a set of eight proteins (Table 3). Remarkably, one of these proteins overlapped with our set of vaccine candidates (CSUI_005805). This protein had no annotated function, but showed a very high expression level (FPKM = 10,244) (Fig.7B) and was *C. suis*-specific, according the OrthoMCL orthology assignment, making it a very attractive vaccine candidate. The protein was predicted to be short (389 amino acids), with a molecular weight of 42 kDa and encoded by a single-exon gene located on contig 2816. To further characterise this protein we analysed its sequence using Phobius (Kall et al., 2007), which identified two transmembrane domains interspersed by a short cytoplasmic region and followed by a longer extracellular tail (Fig.9A). Screening of this protein with the B-cell epitope predictor from the IEDB Analysis Resource tools (Larsen et al., 2006) revealed the presence of several putative epitopes along the sequence (Fig.9B). No additional information about the function of CSUI_005805 was currently available, as this protein lacks orthologs in other organisms. By virtue of all its features such as a high Vacceed score, high expression, species specificity and in vitro immunoreactivity, we conclude that the CSUI_005805 protein constitutes an attractive vaccine candidate for further experimental testing.

## 4 Discussion

In this study, we sequenced, assembled and annotated the genome of *C. suis,* an apicomplexan species of worldwide veterinary relevance. We used this new resource to predict a panel of putative vaccine candidate proteins, which hopefully will serve to develop a novel subunit vaccine. We performed this analysis by combining in silico predictors of protein immunogenicity with transcription and comparative genomics data.

Comparison with publicly accessible genome sequences for other coccidian species identified a relatively large assembly for *C. suis*, with only *S. neurona* found to be bigger. To understand whether this discrepancy was due to expansion of intergenic regions within the *C. suis* genome, we compared the length of genic and intergenic regions in *C. suis* and *T. gondii* and found no significant difference in proportions between the two species (*C. suis* intergenic = 26.3 Mb, genic = 57.4 Mb; *T. gondii* intergenic = 16.5 Mb, genic = 49.2 Mb; Fisher’s Exact Test *P* = 0.4629). Similarly, the proportions of exon to intron lengths were also not significantly different between the two species (*C. suis* exons = 34.6 Mb, introns = 23.5 Mb; *T. gondii* exons = 29.7 Mb, introns = 19.9 Mb; Fisher’s Exact Test *P* = 1). Finally, repetitive regions were also not responsible for the difference in genome size (*C. suis* repetitive = 5.8 Mb, *C. suis* non-repetitive = 77.8 Mb, *T. gondii* repetitive = 2.6 Mb, *T. gondii* non-repetitive = 63.1 Mb; Fisher’s Exact Test *P* = 0.5371). However, we caution that different genome assembly technologies and annotation strategies for different species might bias the comparison of genomic features among assemblies. Features such as average GC content were also consistent with those reported for other coccidians (Kissinger and DeBarry, 2011). Alignment of the *C. suis* contigs to assemblies representing the closest coccidian relatives showed that less than 30% of the *C. suis* assembly could be aligned to *T. gondii* or *N. caninum*. It has previously been shown that 90% of the *N. caninum* contig base pairs could be aligned to the *T. gondii* assembly, indicating a greater evolutionary divergence between *C. suis* and *T. gondii*. The next step will be to estimate the evolutionary divergence in millions of years between *C. suis* and its sister species.

Our annotation of the predicted *C. suis* transcriptome suggests more than 11,000 genes, considerably higher than most other coccidians, but consistent with the larger genome size. We evaluated the quality of our annotation using a range of metrics and found 86% of the core eukaryotic genes to be present. Notably, if we exclude core eukaryotic genes that are absent in the whole coccidian clade, thus also expected to be absent in *C. suis*, this proportion rises to 93%. Expression data validated 90% of the gene models, pointing to a high degree of completeness and reliability of the gene structures. Additionally, we performed a gene-by-gene manual curation, which greatly enhanced the quality of the annotation, by increasing the concordance with transcriptional evidence by almost 20% and the number of genes with UTRs by ∼30%. The larger number of genes predicted compared with most other coccidians might be a consequence of more orphan genes (Martens et al., 2008), supported by the fact that more than 40% of the *C. suis* genes could not be assigned to any orthologous group. Alternatively, fragmented gene models might have resulted in an overestimation of gene number. However, gene numbers in other coccidians might also have been underestimated since most recent RNA-Seq based annotations (Ramaprasad et al., 2015) have not yet been incorporated into ToxoDB. In our annotation, most of the genes identified (99%) had coding potential. While non-coding RNAs were not explicitly annotated, it is likely that polyadenylated non-coding RNA (Matrajt, 2010), such as long non-coding RNAs (lincRNAs), constitute a minor fraction of the gene catalogue of *C. suis*, as also previously shown for *T. gondii* (Hassan et al., 2012).

Another feature specific to the apicomplexan clade is the presence of the apicoplast organelle in most of its members, except the gregarine-like *Cryptosporidium* (Zhu et al., 2000). Two contigs were found to contain most the *C. suis* apicoplast genome, confirming the presence of this organelle in this species. Comparison with *T. gondii* revealed a high level of conservation for the *C. suis* apicoplast genes, although some genes contained premature stop codons, implying a recent pseudogenisation event. This phenomenon has also been described in *T. gondii* (Sato, 2011), where it was suggested that internal stop codons might be interpreted as tryptophan coding by the translation machinery. The absence of transcripts derived from these genes within the RNA-Seq data preclude confirmation for *C. suis* since transcription may simply have been low in the single merozoite lifecycle stage sampled. Consistent with these results, *T. gondii* orthologs of *C. suis* apicoplast genes also had very low expression levels in 3 days p.i. merozoites according to the expression data from the ToxoDB database (Hehl et al., 2015) (Supplementary Table S3).

Screening the predicted *C. suis* proteome, 1,169 putative vaccine candidates were identified using the software Vacceed. We further characterised the candidates according to function, conservation, expression and overlap with candidates that had been tested in other coccidians. Most of the candidates were annotated as of unknown function and remarkably many had no orthologs in other coccidian species. Such diversity might be due to accelerated evolution of proteins that interact with the immune system of the host, as formerly reported for other apicomplexan species (Templeton et al., 2004, Jackson et al., 2014, Blake et al., 2015). Vaccine candidate proteins involved in host interaction and invasion such as apicomplexan-specific secretory organelles proteins, surface antigens and cell adhesion proteins were highly expressed in merozoites, as might have been expected given their function in the *C. suis* lifecycle. Interestingly, when vaccine candidates identified in other coccidian species were compared, only 22% of the candidates with orthologs in *C. suis* had a high Vacceed score. To understand why some known candidates had a low score in *C. suis* we looked at the partial scores from the various tools that constitute the Vacceed pipeline. Many proteins had very low partial scores, indicating the absence of specific signals for membrane, secretion or MHC1-binding epitopes. Additionally, when we looked at protein domains from the InterPro database we did not find any domain related to membrane, secretion or interaction with the immune system (data not shown). This indicates that membrane-related signals might not always be required features for an anticoccidial vaccine candidate. A relatively high proportion (43%) of candidates identified in other coccidians had no orthologs in *C. suis*. By looking at the phylogenetic patterns of these proteins, these candidates were found to be either specific to *E. tenella* (gam56, gam82, GX3262, GX3264, MA16) or they were proteins that originated just before the split of *N. caninum*, *H. hammondi* and *T. gondii*, mostly rhoptry kinases, dense granules and some surface antigens of the SRS family. These results also reflect the likely fast evolution of immune-related proteins. Finally, by overlapping the vaccine candidates obtained by Vacceed with proteins identified from an immunoblot experiment of pig serum, we pinpointed a promising new vaccine candidate corresponding to a 42 kDa transmembrane protein with unknown function (CSUI_005805). However, only a few proteins were recognised by positive sera from infected piglets. More sensitive detection methods or increased amounts of proteins on the gel would certainly reveal more positive spots. To further confirm the usefulness of candidates identified by reverse vaccinology and immunoblotting, recombinant proteins must be generated and characterised in vitro and in vivo in further experiments

In summary, we combined reverse vaccinology with transcriptomics and comparative genomics to identify a list of vaccine candidate proteins for further experimental testing. In order to restrict this set of candidates, new indicators of immunogenicity could be incorporated into the Vacceed pipeline, which is feasible due to the modularity of this tool. Studies on putatively immunogenic proteins of *C. suis* will also greatly enhance our understanding of the immune mechanisms underlying protection (or immunopathology) in porcine cystoisosporosis. Lastly, the genome and annotation of *C. suis* constitute a new step in the genomic era of apicomplexans. As the genus *Cystoisospora* can also be found in other hosts such as dogs, cats and humans, we anticipate that these resources will help to unravel the evolutionary mechanisms of host specificity in apicomplexan parasites.

## Figures and Tables

**Table 1:** Comparison of genome features among *Cystoisospora suis* and its closest relatives from the subclass Coccidia. Organisms are sorted according to phylogenetic position (Fig. 2). Data for other coccidia were extracted from ToxoDB version 24 (for *Sarcocystis neurona,* version 28).

Feature	*Eimeria tenella*	*Sarcocystis neurona*	*Cystoisospora suis*	*Neospora caninum*	*Hammondia hammondi*	*Toxoplasma gondii*
Genome size (Mb)	51.9	128	83.6	59.1	67.7	65.7
GC content (%)	55.0	51.2	50.0	52.8	61.0	48.5

**Table 2:** Comparison of annotation features among *Cystoisospora suis* and its closest relatives from the subclass Coccidia. Organisms are sorted according to phylogenetic position (Fig. 2). Data for other coccidia were extracted from ToxoDB version 24 (for *Sarcocystis neurona,* version 28).

Feature	*Eimeria tenella*	*Sarcocystis neurona*	*Cystoisospora suis*	*Neospora caninum*	*Hammondia hammondi*	*Toxoplasma gondii*
Genes	8,634	7,089	11,572	7,266	8,176	8,920
Transcripts	8,634	7,089	11,572	7,266	8,176	8,920
Exons	38,606	43,976	47,706	43,092	47,096	49,023
Genes with 5′UTRs	0	0	9,806	0^a^	0	5,699^a^
Genes with 3′UTRs	0	0	8,485	0^a^	0	6,128^a^

a Untranslated regions (UTRs) have been annotated by Ramaprasad et al. (2015), but not yet included in ToxoDB.

**Table 3:** Proteins identified in the immunoblot experiment with pig serum. Vaccine candidates are highlighted in gray.

FPKM, fragments per kilobase of exon per million reads.

## Appendix A Supplementary data

Supplementary Table S1 Read mapping statistics from the RNA-Seq dataset used in this study.

Supplementary Table S2 Read mapping statistics from the DNA-Seq dataset used in this study.

Supplementary Table S3 List of genes in the *Cystoisospora suis* apicoplast genome.

Supplementary Table S4 List of eukaryotic core genes that are absent in *Cystoisospora suis* due to gene loss before the split of the coccidian clade.

Supplementary Table S5 Stringent list of *Cystoisospora suis* vaccine candidates.
