# Supplementary material for: The genome of the protozoan parasite Cystoisospora suis and a reverse vaccinology approach to identify vaccine candidates
Source: Int J Parasitol. 2017 Mar;47(4):189–202. doi: 10.1016/j.ijpara.2016.11.007 (PMC5354109; doi:10.1016/j.ijpara.2016.11.007)
Supplement: Supplementary Table S1 — Read mapping statistics from the RNA-Seq dataset used in this study. [file mmc1.docx]

**Supplementary Table S1.** Read mapping statistics from the RNA-Seq dataset used in this study.

| **Dataset** | **No. of read pairs** |
| --- | --- |
| Before assembly | 33,784,376 |
| Mapped to the combined reference (pig + *Cystoisospora suis*) | 32,023,676 |
| Mapped only to pig | 276,963 |
| Mapped only to *C. suis* | 31,746,713 |
