# Supplementary material for: The genome of the protozoan parasite Cystoisospora suis and a reverse vaccinology approach to identify vaccine candidates
Source: Int J Parasitol. 2017 Mar;47(4):189–202. doi: 10.1016/j.ijpara.2016.11.007 (PMC5354109; doi:10.1016/j.ijpara.2016.11.007)
Supplement: Supplementary Table S2 — Read mapping statistics from the DNA-Seq dataset used in this study. [file mmc2.docx]

# Supplementary Table S2. Read mapping statistics from the DNA-Seq dataset used in this study.

| **Dataset** | **No. of reads** |
| --- | --- |
| Before assembly | 84,370,064 |
| Used for genome assembly | 80,264,018 |
