# Supplementary material for: The genome of the protozoan parasite Cystoisospora suis and a reverse vaccinology approach to identify vaccine candidates
Source: Int J Parasitol. 2017 Mar;47(4):189–202. doi: 10.1016/j.ijpara.2016.11.007 (PMC5354109; doi:10.1016/j.ijpara.2016.11.007)
Supplement: Supplementary Table S4 — List of eukaryotic core genes that are absent in Cystoisospora suis due to gene loss before the split of the coccidian clade. [file mmc4.docx]

**Supplementary Table S4.** List of eukaryotic core genes that are absent in *Cystoisospora suis* due to gene loss before the split of the coccidian clade.

| **KOG ID** | **Function** |
| --- | --- |
| KOG0142 | Isopentenyl-diphosphate Delta-isomerase (secondary metabolites biosynthesis, transport and catabolism) |
| KOG0784 | Isocitrate dehydrogenase, gamma subunit (amino acid transport and metabolism) |
| KOG0788 | S-adenosylmethionine decarboxylase proenzyme (amino acid transport and metabolism) |
| KOG1185 | Acetolactate synthase (amino acid transport and metabolism, Coenzyme transport and metabolism) |
| KOG1211 | Amidases (translation, ribosomal structure and biogenesis) |
| KOG1393 | Synthase (lipid transport and metabolism) |
| KOG1535 | Fumarylacetoacetate hydrolase (secondary metabolites biosynthesis, transport and catabolism) |
| KOG1562 | Spermidine synthase (amino acid transport and metabolism) |
| KOG1566 | Calcium binding protein (function unknown) |
| KOG1712 | Adenine phosphoribosyltransferase (nucleotide transport and metabolism) |
| KOG2531 | Sugar (pentulose and hexulose) kinases (Carbohydrate transport and metabolism) |
| KOG2555 | AICAR transformylase/IMP cyclohydrolase/methylglyoxal synthase (nucleotide transport and metabolism) |
| KOG2638 | UDP-glucose pyrophosphorylase (carbohydrate transport and metabolism) |
| KOG2833 | Mevalonate pyrophosphate decarboxylase (lipid transport and metabolism) |
| KOG3284 | Vacuolar sorting protein VPS28 (intracellular trafficking, secretion and vesicular transport) |
| KOG3463 | Transcription initiation factor IIA, gamma subunit (transcription) |
| KOG3855 | Monooxygenase involved in coenzyme Q (ubiquinone) biosynthesis (coenzyme transport and metabolism, energy production and conversion) |
